# AI-Based 3D Modeling Strategies for Civil Infrastructure: Quantitative Assessment of NeRF and Photogrammetry

**DOI:** 10.3390/s26030852

**Published:** 2026-01-28

**Authors:** Edison Atencio, Fabrizzio Duarte, Fidel Lozano-Galant, Rocio Porras, Ye Xia

**Affiliations:** 1 School of Civil Engineering, Pontificia Universidad Católica de Valparaíso, Avenida Brasil 2147, Valparaíso 2362804, Chile; fabrizzio.duarte.s@mail.pucv.cl; 2 Department of Civil and Building Engineering, Universidad de Castilla-La Mancha, Av. Camilo Jose Cela s/n, 13071 Ciudad Real, Spain; fidel.lozanogalant@uclm.es; 3 School of Civil Engineering, University of Castilla-La Mancha, 13071 Ciudad Real, Spain; rocio.porras@uclm.es; 4 Department of Bridge Engineering, Tongji University, 1239 Siping Rd., Shanghai 200092, China

**Keywords:** 3D modeling, point cloud accuracy, design science research, NeRF neural radiance fields, UAV unmanned aerial vehicle imagery, infrastructure reconstruction

## Abstract

Three-dimensional (3D) modeling technologies are increasingly vital in civil engineering, providing precise digital representations of infrastructure for analysis, supervision, and planning. This study presents a comparative assessment of Neural Radiance Fields (NeRFs) and digital photogrammetry using a real-world case study involving a terrace at the Civil Engineering School of the Pontificia Universidad Católica de Valparaíso. The comparison is motivated by the operational complexity of image acquisition campaigns, where large image datasets increase flight time, fieldwork effort, and survey costs. Both techniques were evaluated across varying levels of data availability to analyze reconstruction behavior under progressively constrained image acquisition conditions, rather than to propose new algorithms. NeRF and photogrammetry were compared based on visual quality, point cloud density, geometric accuracy, and processing time. Results indicate that NeRF delivers fast, photorealistic outputs even with reduced image input, enabling efficient coverage with fewer images, while photogrammetry remains superior in metric accuracy and structural completeness. The study concludes by proposing an application-oriented evaluation framework and potential hybrid workflows to guide the selection of 3D modeling technologies based on specific engineering objectives, survey design constraints, and resource availability while also highlighting how AI-based reconstruction methods can support emerging digital workflows in infrastructure monitoring under variable or limited data conditions.

## 1. Introduction

Three-dimensional (3D) modeling has become a key enabler in civil engineering, supporting applications such as construction monitoring, structural assessment, volumetric analysis, and infrastructure documentation [1,2,3]. Accurate digital reconstructions allow engineers to analyze existing assets, detect geometric deviations, and support decision-making within increasingly digitalized Architecture, Engineering, and Construction (AEC) workflows.

Digital photogrammetry remains one of the most widely adopted approaches for 3D reconstruction, relying on Structure-from-Motion (SfM) and Multi-View Stereo (MVS) algorithms to generate dense point clouds and textured meshes from overlapping images. Commercial solutions such as Bentley’s iTwin Capture Modeler are commonly used in professional practice because they deliver high geometric fidelity when sufficient imagery and metadata are available [4]. However, photogrammetric workflows typically require extensive image datasets, careful flight planning, and significant computational resources. As a result, large datasets often translate into longer UAV flight times, increased fieldwork complexity, and higher operational costs, which can limit survey scalability under real-world constraints.

Neural Radiance Fields (NeRFs), initially introduced by [5], represent a fundamentally different paradigm for image-based 3D reconstruction. By learning a continuous volumetric representation of a scene through deep neural networks, NeRF enables photorealistic rendering from relatively sparse image sets. Recent developments, including Nerfacto within the Nerfstudio framework and Instant-NGP [6], have substantially improved training efficiency and rendering quality, expanding the feasibility of NeRF for real-world applications.

Compared to traditional techniques such as photogrammetry or LiDAR, NeRF exhibits advantages in handling illumination variability and challenging surface properties, and can produce visually coherent reconstructions even from incomplete datasets [7]. These characteristics have motivated increasing interest in NeRF across domains such as robotics, virtual and augmented reality, cultural heritage, and, more recently, civil infrastructure modeling for inspection, documentation, and digital twin development.

Beyond methodological considerations, image availability has direct operational implications in civil engineering surveys. Reducing the number of required images can enable broader spatial coverage per UAV flight, shorten field acquisition time, simplify mission planning, and lower data management and processing costs. From this perspective, evaluating whether alternative reconstruction paradigms can maintain acceptable performance with fewer images is a practical engineering question that directly affects survey design, resource allocation, and the feasibility of large-scale or time-sensitive inspections.

Although reconstruction under limited-data conditions has been extensively investigated from an algorithmic standpoint, most existing studies focus on methodological advances, synthetic benchmarks, or isolated performance metrics. Comparatively little attention has been paid to application-driven analyses that relate reconstruction behavior to operational constraints typical of engineering practice, where mature software tools are used, and data-acquisition efficiency is a primary concern.

This study addresses this gap through a systematic, condition-oriented comparison of Neural Radiance Fields and digital photogrammetry under progressively reduced image availability. Rather than proposing new reconstruction algorithms, the work analyzes reconstruction behavior using representative and widely adopted implementations—Nerfacto (Nerfstudio) and iTwin Capture Modeler—to reflect realistic engineering workflows. A terrace at the Civil Engineering School of the Pontificia Universidad Católica de Valparaíso (PUCV) is used as a case study, based on an initial UAV dataset of 475 images that is progressively reduced in 10% increments. The resulting models are evaluated for visual fidelity, point cloud geometric accuracy (measured with CloudCompare v2.13.2, July 2024 release), and processing time.

By relating reconstruction outcomes to both data availability and operational considerations, this work provides application-relevant insights into the trade-offs between robustness, geometric fidelity, field efficiency, and computational effort. The main contribution lies in the proposed evaluation framework and interpretative analysis, which are methodologically transferable across platforms and support informed decision-making in time- and resource-constrained civil engineering contexts [8,9].

The remainder of this article is organized as follows. Section 2 presents the research background. Section 3 describes the methodology and experimental design. Section 4 reports the empirical results. Section 5 discusses the findings in relation to existing literature and civil engineering applications, and Section 6 concludes the paper and outlines directions for future research on hybrid modeling strategies and NeRF optimization.

## 2. Background

### 2.1. Advances in 3D Reconstruction Technologies: From Photogrammetry to Neural Radiance Fields

Three-dimensional (3D) modeling is a cornerstone of modern civil engineering, enabling accurate representation, supervision, and planning of infrastructure projects. By creating digital replicas of physical assets, 3D models support structural analysis, construction monitoring, defect detection, and design optimization. Among image-based approaches, photogrammetry remains one of the most established techniques, relying on geometric principles such as Structure-from-Motion (SfM) and Multi-View Stereo (MVS) to reconstruct dense and metrically accurate models from overlapping images.

UAV-based photogrammetry has been widely adopted for large-scale and complex engineering structures. Studies such as [4] demonstrate that camera calibration, flight geometry, and image overlap significantly influence reconstruction accuracy, highlighting the importance of acquisition parameters when assessing geometric fidelity. In heritage and large-scale documentation contexts, photogrammetry has been combined with MVS and neural texture modeling to enhance visual realism and completeness, outperforming some commercial tools in controlled scenarios [10].

Continuous advances in camera calibration, feature detection, matching algorithms, and bundle adjustment have further strengthened photogrammetry’s ability to generate reliable point clouds and meshes for engineering applications [11]. Its main strength lies in explicit geometric reconstruction, which enables high metric accuracy and structural interpretability, particularly when camera poses are well constrained.

Despite these advantages, photogrammetry exhibits limitations when applied to reflective, translucent, or texture-poor surfaces, such as glass, water bodies, or uniform concrete elements [12]. These conditions challenge feature detection and matching, often resulting in incomplete or noisy reconstructions. As noted in [13], such limitations are particularly relevant for construction quality assessment, where alternative image-based strategies using camera poses rather than dense point clouds can still achieve high precision.

Photogrammetric workflows are also sensitive to suboptimal acquisition conditions, including poor lighting, shadows, or sparse image coverage. In these cases, reconstruction quality may degrade through fragmented point clouds, blurred textures, or mesh gaps. Moreover, the multi-stage nature of photogrammetry—encompassing feature extraction, matching, triangulation, depth estimation, and meshing—introduces cumulative error sources. It increases processing time, which can be critical in time-sensitive or resource-constrained projects [14].

Neural Radiance Fields (NeRFs), introduced by [5], represent a fundamentally different reconstruction paradigm. Instead of explicitly estimating discrete geometry, NeRFs learn a continuous volumetric representation of a scene using deep neural networks, enabling photorealistic view synthesis from sparse image inputs. This implicit representation enables NeRFs to capture complex optical effects, such as reflectivity, translucency, and subtle shading, that are difficult for conventional methods to reproduce [15,16].

Unlike photogrammetry, NeRFs regress color and volume density along camera rays rather than reconstructing explicit point clouds or meshes. This formulation enables robust interpolation of novel viewpoints, even with uneven or sparse image distributions [17]. However, early NeRF implementations suffered from high computational demands and limited geometric accuracy, restricting their applicability in metric-sensitive engineering tasks [18,19]. Recent developments in architecture and training strategies, including hybrid approaches incorporating depth priors and volumetric editing tools, have progressively improved NeRF performance in terms of speed, robustness, and practical usability [18,20,21].

Emerging applications in augmented reality, embedded systems, and real-time visualization further demonstrate the growing operational feasibility of NeRFs for infrastructure inspection, construction monitoring, and digital twin development [7,15]. Collectively, these advances suggest that NeRFs are transitioning from experimental techniques toward viable complements to traditional photogrammetry and LiDAR-based workflows.

### 2.2. Comparison Between NeRF and Photogrammetry

With increasing interest in applying NeRFs in civil engineering, several comparative studies have evaluated their performance relative to conventional photogrammetry. These comparisons generally focus on three aspects: visual fidelity, metric accuracy, and computational efficiency. A consistent finding across the literature is that NeRF-based reconstructions tend to outperform photogrammetry in terms of visual realism, particularly under limited image availability or adverse conditions such as reflective surfaces, texture-less areas, and occlusions [22,23]. In cultural heritage and complex surface scenarios, NeRF has been shown to generate visually coherent reconstructions from sparse UAV imagery where photogrammetry struggles due to insufficient texture gradients [24,25].

In contrast, photogrammetry continues to demonstrate superior metric accuracy and sharper geometric boundaries. Quantitative comparisons between SfM-based pipelines and NeRF reconstructions indicate that photogrammetry produces more precise object edges and scale-consistent models, mainly when supported by ground control points or well-constrained camera geometry [26,27]. This explicit geometric formulation remains a key advantage for engineering applications that require reliable dimensional measurements and structural interpretability [28].

Within construction-related scenarios, NeRF has been explored for progress monitoring, visual documentation, and scan-to-BIM support [29,30]. While NeRF enables rapid visualization and flexible viewpoint generation for inspection and communication purposes [31], photogrammetry remains the preferred method for detailed as-built modeling and structural assessment tasks where metric accuracy is critical [28,32]. As a result, hybrid workflows have been proposed in which NeRF provides immersive visualization and interpolated views, while photogrammetry or laser scanning supplies the underlying metric geometry [11,12].

From a computational perspective, photogrammetry—despite its multi-stage nature—can be efficient when deployed with optimized pipelines and GPU acceleration. Nevertheless, recent NeRF architectures, such as Instant Neural Graphics Primitives (Instant-NGP), Multi-Plane NeRF (MP-NeRF), and Flexible and Fast NeRF (F2-NeRF), have significantly reduced training and rendering times, achieving near real-time performance on commodity hardware [15,33,34]. These developments improve NeRF’s suitability for field-oriented applications, particularly in scenarios involving blurred imagery, low-light conditions, or flexible camera trajectories [34].

A further distinction between the two paradigms concerns scalability and integration within established engineering workflows. Photogrammetry naturally integrates with Computer-Aided Design (CAD) and Building Information Modeling (BIM) environments through exportable meshes and point clouds. NeRF outputs, by contrast, are volumetric and less directly compatible with industry-standard tools. However, emerging NeRF-to-mesh conversion techniques, semantic segmentation approaches for BIM integration, and hybrid mesh-enhanced methods are progressively addressing these limitations [35,36].

Overall, the literature indicates that NeRF excels at visual coherence and flexibility under constrained acquisition conditions, whereas photogrammetry remains superior for applications that demand high geometric accuracy and structural reliability. Consequently, method selection should be guided by application requirements, with hybrid strategies offering a promising approach for combining the strengths of both techniques [37].

### 2.3. Emerging Directions and Relevance for Civil Engineering Applications

Recent research highlights several emerging directions that enhance NeRF’s relevance to civil engineering. One prominent trend involves integrating auxiliary data sources—such as LiDAR, RGB-D cameras, and event-based sensors—to provide geometric constraints and improve scale consistency in NeRF reconstructions [18,20,38,39]. These hybrid configurations reduce NeRF’s dependence on dense image coverage and enhance geometric interpretability.

Another growing area of interest is uncertainty quantification in NeRF-based reconstructions. In engineering contexts where decisions rely on 3D data quality, probabilistic confidence measures are increasingly necessary. Approaches such as ProbNeRF and Conditional-Flow NeRF aim to estimate reconstruction uncertainty alongside visual outputs, improving the reliability of NeRF-generated models for decision-making tasks [40].

Scalability has also received increasing attention, particularly for large-scale infrastructure and urban environments. Architectures such as Block-NeRF and Urban Radiance Fields introduce spatial partitioning and mesh-based primitives that enable real-time interaction, reduced memory requirements, and incremental updates—capabilities that are essential for future digital twin systems [41].

Specific civil engineering applications, including infrastructure inspection, construction monitoring, and progress tracking, have already demonstrated the practical value of NeRF for rapid field documentation [42]. Compatibility with UAVs, mobile devices, and mixed-reality platforms such as HoloLens enables on-site 3D visualization with minimal setup, supporting faster situational awareness for engineers and site managers [43].

Advances in rendering control, including lighting- and reflectance-aware NeRF variants such as ReNeRF and NeRFactor, further expand NeRF’s potential for material analysis, environmental visualization, and energy-related studies [44,45]. More recent developments, including NeRF-GANs and SceneRF, reflect the evolution of NeRF from purely visual representation techniques toward more intelligent and interactive 3D modeling frameworks [46].

Despite these advances, the reviewed literature reveals a persistent gap from an application-oriented perspective. While numerous studies address algorithmic improvements and benchmark performance under sparse data conditions, there remains limited empirical evidence on how NeRF and photogrammetry behave under progressively reduced image acquisition scenarios typical of real-world engineering surveys. Factors such as restricted access, occlusions, limited flight time, and cost constraints often necessitate reduced datasets, yet their operational impact on reconstruction quality and efficiency remains poorly understood.

This study addresses this gap by conducting a comparative experimental analysis that progressively reduces UAV-captured imagery to evaluate the performance of NeRF and digital photogrammetry under realistic acquisition constraints. Using a Design Science Research Methodology (DSRM), the analysis focuses on reconstruction behavior, trade-offs, and practical usability to support informed technology selection and survey design in civil engineering applications.

## 3. Methodology

This study adopts the Design Science Research Methodology (DSRM) as a guiding framework for structuring the research process. DSRM is particularly suitable for evaluating technological artifacts in real-world contexts, organizing the study into five iterative stages: (1) Problem identification, (2) Objective definition, (3) Artifact design, (4) Implementation, and (5) Evaluation (See Table 1).

As shown in Table 1, the first stage involved a comprehensive literature review of NeRF to identify its main applications, advantages, and limitations in 3D modeling. The targeted objective was to understand the current state of the art and assess NeRF’s potential in civil engineering contexts. This review process also helped identify the most widely used NeRF variants and the evaluation criteria used in previous studies. The full details and results of this literature review are presented in Section 2.2 of the paper.

In the second stage, the research defined specific objectives to evaluate and compare the performance of NeRF using the Nerfacto model within the Nerfstudio platform and photogrammetric application. Photogrammetric reconstructions were performed using widely adopted commercial software solutions available in 2024. iTwin Capture Modeler (2024.1.0, build 24.01.00.560, March 2024 release) was employed as a representative industry-standard tool. Reconstructions were generated using default and vendor-recommended processing settings, without manual parameter fine-tuning, to reflect typical professional workflows and avoid user-induced bias. Standard photogrammetric pipelines were applied, including feature extraction, image alignment, dense point cloud generation, and surface reconstruction. Input images were processed at their original resolution, and no additional pre-processing beyond standard software routines was applied. All processing was performed in the previously described hardware environment, ensuring consistency across experiments. While some internal parameters of proprietary software are not fully accessible, using stable software versions and default configurations ensures that reconstruction outcomes are representative and reproducible within the context of contemporary professional practice.

Three key evaluation criteria were established to guide the comparison: visual quality, processing time, and geometric accuracy. To ensure controlled experimentation, a UAV flight campaign was conducted to capture a dataset of 475 images of a real-world terrace. The photos were taken from varying camera angles and altitudes—184 images at a 90° angle and 15 m altitude, 198 images at 45° and 10 m, and 93 images at 30° and 8 m—following recommendations from established photogrammetric literature [47]. To assess performance under limited-data conditions, the dataset was systematically reduced in 10% increments, producing ten subsets ranging from 100% to 10% of the original images.

In the third phase, a workflow was established to process the image sets and generate 3D models using both NeRF and photogrammetry. The workflow included all necessary steps to ensure the replicability and comparability of results between the two modeling approaches. To use NeRF, it was first required to obtain camera poses for each image. For this purpose, Agisoft Metashape Professional version 2.1.1 was employed, as Nerfstudio requires prior camera parameter estimation.

NeRF-based reconstructions were generated using the Nerfacto pipeline implemented in the Nerfstudio framework, selected as a representative state-of-the-art NeRF implementation commonly adopted in applied reconstruction workflows. The study does not aim to benchmark multiple NeRF variants or optimize hyperparameters, but to analyze reconstruction behavior under application-driven constraints.

Camera poses estimated in Agisoft Metashape were imported into Nerfstudio before training. All models were trained using the default Nerfacto configuration recommended by the framework at the time of the experiments, without manual hyperparameter tuning, to ensure consistency across datasets and reflect typical applied usage scenarios. Training was performed for 30,000 iterations using the Adam optimizer, with an initial learning rate of 1 × 10^−2^ and an exponential decay schedule. A uniform ray sampling strategy and the standard Nerfacto network architecture were employed, with surface normal prediction enabled to improve geometric consistency. All experiments were executed on an NVIDIA RTX 4060 GPU under Windows 11.

In parallel, iTwin Capture Modeler processed the same image sets directly, leveraging embedded geolocation metadata to generate photogrammetric models without additional preprocessing. In parallel, iTwin Capture Modeler processed the same image sets directly, relying on the embedded image metadata and standard vendor-recommended processing settings. No manual image preprocessing steps—such as radiometric enhancement, image filtering, resolution rescaling, masking, or external calibration adjustments—were applied before photogrammetric reconstruction.

In the fourth stage, once the workflow was defined, the modeling process was implemented across all image configurations. Each dataset was processed independently through both Nerfstudio (NeRF) and iTwin Capture Modeler (photogrammetry), generating respective 3D models. This enabled a direct performance comparison under progressively constrained input conditions.

The final stage focused on assessing the results using the three previously defined criteria. Processing time was recorded for each experiment. The generated point clouds and meshes were visually and metrically evaluated using Nerfstudio’s internal tools and CloudCompare. Comparative graphs and tables were created to analyze the trade-offs between visual realism, accuracy, and computational efficiency across both approaches.

Although the reconstructed models are not treated as absolute ground-truth representations, the use of Ground Control Point (GCP)-based spatial control ensures internal geometric consistency. It enables reliable relative comparisons between reconstruction approaches.

**Figure 1:**
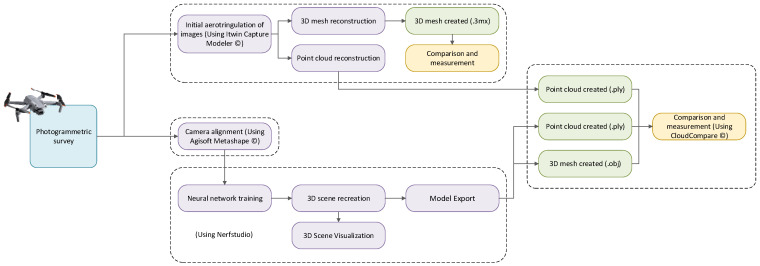
Work methodology.

To ensure replicability and comparability across technologies, a dual processing workflow was established: one for NeRF-based modeling and the other for digital photogrammetry. The complete methodology is summarized in Figure 1.

In the NeRF-based approach, camera poses were first computed using Metashape to obtain accurate spatial alignment from the input images. These poses, along with the corresponding calibration data, were then fed into Nerfstudio using the Nerfacto pipeline, where normal prediction was enabled to improve surface definition. The outputs—dense point clouds and mesh reconstructions—were exported using Poisson surface reconstruction to convert volumetric data into usable mesh geometry.

In the photogrammetric workflow, the same image sets were imported directly into iTwin Capture Modeler, which automatically processed them using embedded georeferencing metadata. The software generated point clouds and high-resolution textured meshes through its integrated Structure-from-Motion and Multi-View Stereo algorithms. The results were exported in .3mx format for visualization and .obj for subsequent geometric evaluation.

### Case Study and Data Acquisition

**Figure 2:**
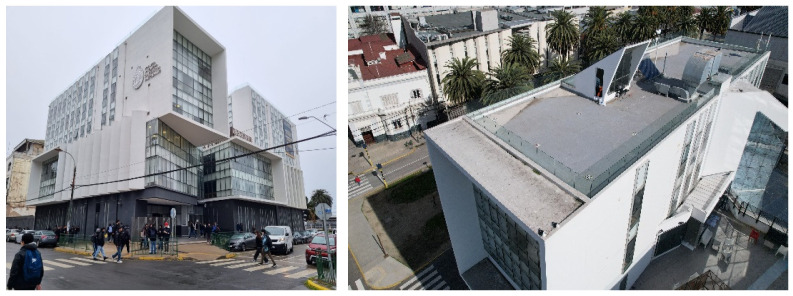
Civil Engineering School at the Pontificia Universidad Católica de Valparaíso (PUCV), Chile.

The selected case study was the terrace of the Civil Engineering School at the Pontificia Universidad Católica de Valparaíso (PUCV), Chile, shown in Figure 2. This location offers accessibility and features reflecting complex-textured surfaces, which pose challenges for the 3D reconstruction technique.

For the photogrammetric flight, a DJI Air 2S drone (Da-Jiang Innovations Science and Technology Co., Ltd., Shenzhen, China) was used. This equipment features a 20 MP camera (5472 × 3648 resolution); front, rear, top, and bottom sensors; and a sensor size of 13.13 × 8.76 mm. The drone has a maximum flight speed of 19 m/s, a maximum battery life of 30 min, and a focal length of 8.8 mm. A total of 475 images were captured at various angles and altitudes to ensure rich geometric information: 184 images at 90° from 15 m, 198 at 45° from 10 m, and 93 at 30° from 8 m. All images were captured with 90% overlap to comply with photogrammetric best practices.

All computational tasks were performed on a desktop workstation running Windows 11, equipped with an Intel Core i7 (11th Gen) processor, 40 GB of RAM, and an NVIDIA RTX 4060 GPU. This setup provided sufficient resources to process high-resolution image sets and train NeRF models efficiently while ensuring fair performance comparisons between the two reconstruction methods.

To provide geometric control and scale consistency, six GCPs were distributed across the terrace area and measured using a Spectra Precision Focus 2 series total station. These GCPs served as high-accuracy reference points for spatial alignment and scaling. The same set of GCPs was consistently used across all reconstruction experiments, ensuring comparable geometric conditions between NeRF-based and photogrammetric models.

To simulate limited data availability, the complete image dataset was progressively reduced in 10% increments, yielding 10 subsets ranging from 100% to 10% of the original images (see Table 2). Images were removed at regular intervals to maintain even spatial distribution and prevent biased coverage gaps. Each subset was processed independently using the two reconstruction workflows.

## 4. Results

Once all the datasets to be used have been defined, the models are generated following the established workflow. First, point clouds are exported in “.ply” format from both software platforms. In the case of Nerfacto, models are trained with normal prediction enabled, allowing for the subsequent export of higher-quality meshes using Poisson surface reconstruction, as recommended by Nerfstudio on its official website. Additionally, when exporting point clouds with Nerfacto, the model’s real-world coordinates are preserved. Table 3, Table 4, Table 5, Table 6, Table 7, Table 8, Table 9, Table 10, Table 11 and Table 12 present the experimental results from progressively reducing the image dataset, comparing the performance of NeRF and photogrammetry in generating 3D models under varying data availability. Each table corresponds to a specific experiment. For each case, point cloud visualizations and processing times were recorded, allowing for a comparative analysis of visual completeness and computational efficiency.

A comparative analysis was conducted to evaluate the performance of iTwin Capture Modelerand Nerfacto across four key criteria: processing time, visual quality, geometric accuracy, and point cloud density.

**Figure 3:**
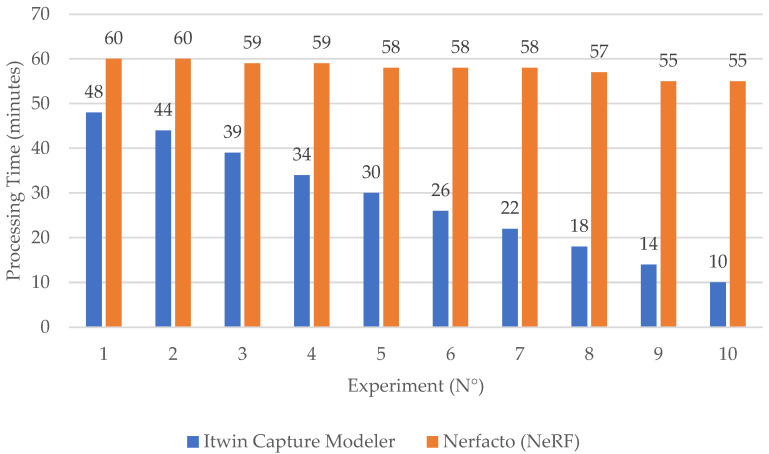
Comparative graph of the time required to generate point clouds in .ply format.

Processing time was measured from image import to the export of point clouds or meshes. As shown in Figure 3, iTwin Capture Modeler consistently outperformed Nerfacto in terms of speed, with a clear linear decrease in processing time as the number of input images decreased. This scalability demonstrates photogrammetry’s efficiency under data-constrained conditions. In contrast, Nerfacto’s processing time remained largely constant across all experiments, indicating fixed training demands regardless of dataset size. This lack of scalability reflects the computational cost of neural network-based scene reconstruction:

In contrast, Nerfacto’s processing time remains relatively constant across experiments, despite the decreasing image count. While this suggests limited scalability in terms of speed, it also reflects the fixed computational requirements of neural training. However, the quality of NeRF outputs declines noticeably in low-data conditions, especially in terms of geometric completeness. These results underscore the strengths of photogrammetry for high-accuracy modeling while highlighting NeRF’s value for fast, visually realistic reconstructions when data or time is limited.

Visual quality was assessed through the inspection of point cloud outputs using the native mesh viewers of both software platforms. In the initial experiments (100% to 70% of the original dataset), Nerfacto produced photorealistic results with smooth transitions and vivid textures, leveraging its volumetric rendering and view-dependent synthesis. However, as image count dropped below 60%, visible gaps, artifacts, and geometric distortions became increasingly prominent—especially in occluded or complex areas. iTwin Capture Modeler, while producing slightly less vivid textures, delivered structurally complete and topologically consistent models across all image subsets, owing to its reliance on geometric triangulation rather than learned appearance.

Geometric accuracy was evaluated using CloudCompare, where models were scaled and compared against real-world measurements. Since NeRF models lack inherent scale, a correction factor was applied based on known distances. Despite this adjustment, Nerfacto reconstructions showed greater spatial inconsistencies, particularly under low-data conditions, where artifacts and incorrect volumes appeared. In contrast, iTwin Capture Modeler consistently generated dimensionally accurate outputs, reinforcing its suitability for engineering-grade applications.

To quantify these differences, meshes exported from both platforms were analyzed. For iTwin Capture Modeler, real-world units were preserved in the mesh, allowing for direct measurements in meters. In contrast, Nerfstudio exports lacked absolute scale, requiring the use of CloudCompare’s default units and the application of a scaling factor. This factor was computed for each model individually by dividing a known real-world length by the corresponding model-derived length, and was then applied uniformly to all subsequent measurements from that experiment.

**Figure 4:**
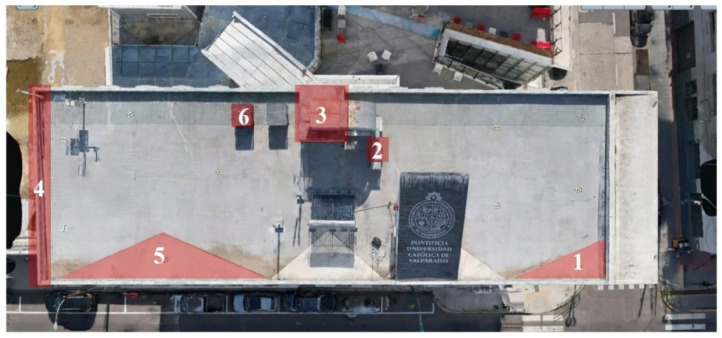
Measured elements to calculate models’ precision.

**Figure 5:**
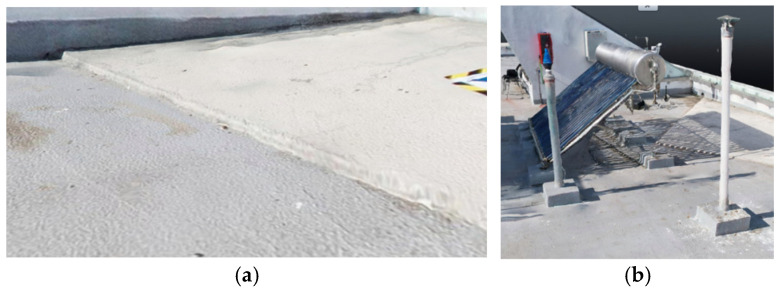
Capacity to model small elements in first experiment. (**a**) iTwin Capture Modelerand. (**b**) Nerfacto.

The comparison focused on a set of predefined measurement elements—termed “packs”—distributed across different regions and dimensions of the model (depicted in Figure 4). However, the Nerfacto meshes struggled to represent small-scale or fine-detail volumes accurately, and the model generated in Experiment 10 was excluded due to its poor quality. Nerfacto’s mesh outputs increasingly failed to capture structural continuity under reduced image conditions. The objects selected for comparative analysis are depicted in Figure 4. Figure 5 further illustrate this difference, highlighting how iTwin Capture Modeler successfully reconstructed small elements that Nerfacto either distorted or omitted entirely.

**Figure 6:**
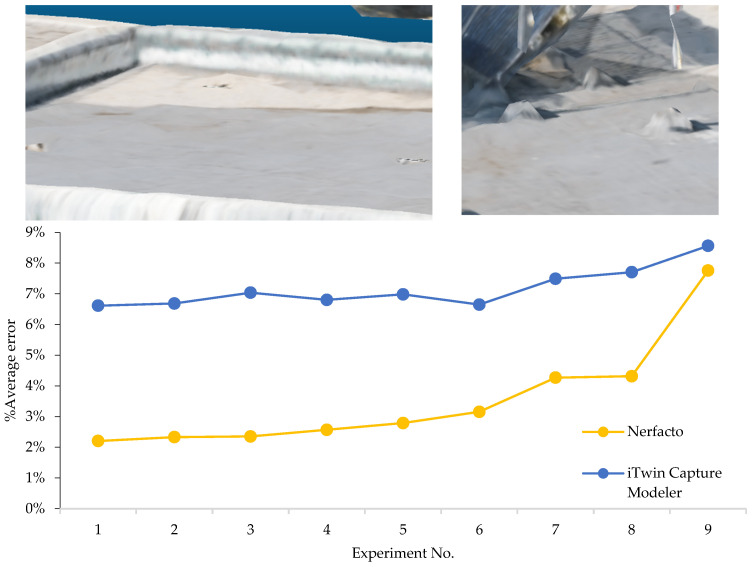
Average error percentages for each method.

For each pack, distances measured on the models were compared to real-world values. In the case of Nerfacto, each measurement was scaled using its respective correction factor. The results for each experiment are summarized in Figure 6, showing average error percentages for each method. While iTwin Capture Modeler exhibited higher average absolute errors than Nerfacto, this may be influenced by the greater mesh detail captured in the photogrammetric outputs, which introduces sensitivity to fine surface variations. Moreover, Nerfacto’s simplified representations tended to preserve overall proportions, even when detail fidelity was low.

This evaluation was repeated for all experiments, and the results are presented in Figure 6, which shows error trends for both methods as image count decreases. As expected, both technologies show increased error with fewer images. iTwin Capture Modeler errors grow steadily, while Nerfacto’s accuracy declines more sharply but remains lower overall in most experiments. Interestingly, Nerfacto achieves better metric precision than photogrammetry even in Experiment 8, despite its visually lower-quality mesh. These findings suggest that while Nerfacto struggles with geometric completeness and detail, it retains proportional consistency across scales, making it potentially useful for certain engineering scenarios—especially under limited data conditions. Further research under controlled and optimized acquisition settings may reveal whether NeRF-based methods can improve both accuracy and detail in tandem.

**Figure 7:**
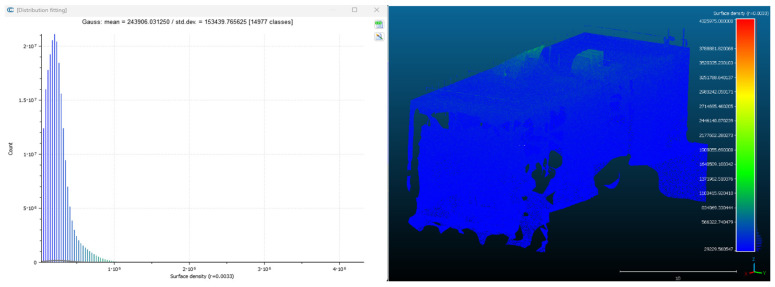
Density of experiment 1 with iTwin Capture Modeler.

**Figure 8:**
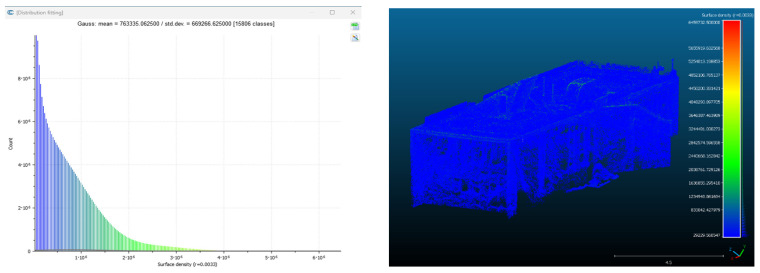
Density of experiment 1 with Nerfacto.

**Figure 9:**
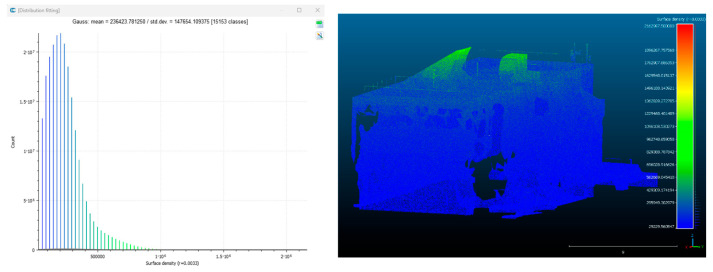
Density of experiment 3 with iTwin Capture Modeler.

**Figure 10:**
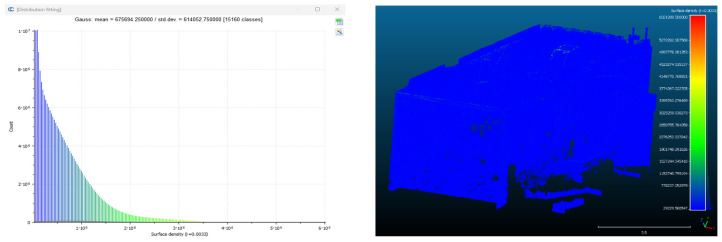
Density of experiment 3 with Nerfacto.

**Figure 11:**
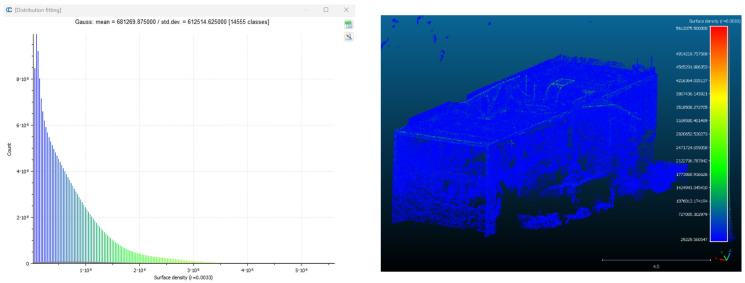
Density of experiment 6 with Nerfacto.

**Figure 12:**
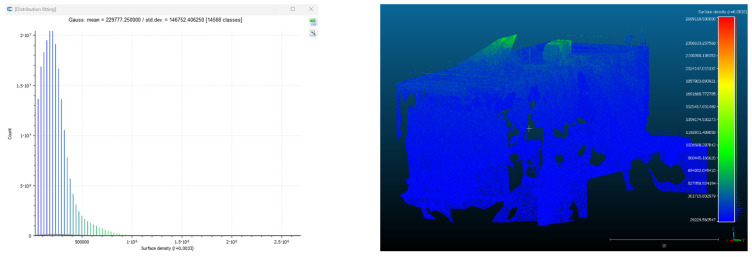
Density of experiment 6 with iTwin Capture Modeler.

Point cloud density was also analyzed using CloudCompare by applying a constant local neighborhood radius of 0.0033 across all models—selected as the average of the software’s suggested values to ensure comparability. Figure 7, Figure 8, Figure 9, Figure 10, Figure 11 and Figure 12 illustrate the distribution of density for each experiment. iTwin Capture Modeler displayed a logical and predictable decrease in density in line with image reduction, maintaining uniform point distribution throughout. Nerfacto, however, exhibited erratic and non-monotonic fluctuations in density, with clustering in unintended regions and voids where structure was expected. Figure 7 summarizes these trends, underscoring the instability of NeRF-derived models when trained on sparse datasets.

In the early experiments (Experiments 1 to 4, using 100% to 70% of the original dataset), NeRF consistently produced visually compelling results, with realistic color rendering and smooth surface transitions. This performance is attributed to NeRF’s inherent design, which prioritizes photorealism through volumetric rendering and view-dependent shading. However, as the image count decreased beyond 60%, the NeRF-generated point clouds began to exhibit evident degradation: spatial discontinuities, surface gaps, and loss of fine detail became progressively more noticeable, especially in occluded or complex regions of the scene. These effects reflect NeRF’s reliance on dense multi-view data for training the neural representation, which becomes less effective when coverage is sparse.

In contrast, iTwin Capture Model demonstrated a more stable behavior across all experiments. While its textures are somewhat less visually refined than those of NeRF, the models remained topologically complete and structurally coherent, even at lower image counts. This robustness is consistent with photogrammetry’s dependence on geometric triangulation rather than learned appearance, allowing it to reconstruct surfaces accurately as long as there is sufficient parallax and image overlap.

These observations, based on empirical visual inspection of exported clouds using native viewers and CloudCompare, support the interpretation that NeRF excels in view-dependent visual realism, whereas photogrammetry remains more reliable for scene completeness and structural continuity—particularly under constrained data conditions.

To assess the geometric reliability of the 3D reconstructions generated by both technologies, a quantitative analysis of point cloud density and spatial consistency was conducted using CloudCompare. After exporting all point clouds, the local point densities were calculated by applying a uniform Local Neighborhood Radius of 0.0033, a value determined as the average of the radii automatically suggested by the software for each model. This standardized radius allowed for fair inter-comparison across the experiments, despite variations in scale and model volume—particularly relevant in the case of NeRF exports, which lack absolute scale by default.

**Figure 13:**
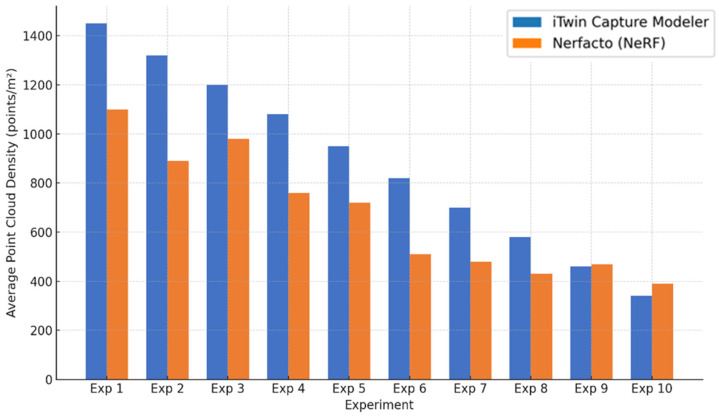
Average point cloud density (points/m^2^) for each experiment using iTwin Capture Modeler and Nerfacto.

To evaluate point cloud density across the experimental conditions without overloading the visual content of this paper, only a subset of representative examples has been selected for inclusion. Specifically, Figure 7, Figure 8, Figure 9, Figure 10, Figure 11, Figure 12 and Figure 13 illustrate the density maps for key experiments that reflect the overall trends: Experiment 1 (full dataset), Experiment 3 (moderate reduction), and Experiment 6 (significant reduction). These were chosen to exemplify typical behavior at different stages of data availability.

The photogrammetry-based models (iTwin Capture Modeler) exhibit a predictable, decreasing trend in point cloud density as the number of input images is reduced from 100% to 10%. This behavior aligns with expectations: fewer images yield fewer matched features, leading to sparser but consistently distributed point clouds. While complete density maps were generated for all experiments using CloudCompare with a constant local neighborhood radius of 0.0033, only this curated selection is presented visually. The rationale for this choice is twofold: first, it enhances readability and clarity; second, the overall patterns of performance divergence between iTwin Capture Modeler and Nerfacto can be observed clearly in the selected examples. For instance, the gradual density loss in photogrammetric models (iTwin Capture Modeler) contrasts with the erratic, often non-monotonic variation in Nerfacto models under similar data reduction.

Quantitative density values for all experiments are summarized in Figure 13, which displays the average points per square meter for each technology across the ten experiments. This allows a comprehensive understanding of performance without the need to examine each individual case visually. This graph illustrates a clear, progressively decreasing trend in point cloud density for iTwin Capture Modeler as image count is reduced—consistent with expected photogrammetric behavior. In contrast, Nerfacto exhibits irregular and non-monotonic fluctuations in density, suggesting instability in model generation under limited data conditions. This variability may result from incomplete scene coverage, uneven point distribution, and geometry inference errors inherent to NeRF when trained with reduced datasets.

Conversely, the models generated by Nerfacto show inconsistent and erratic density behavior across the same sequence of experiments. As shown in Figure 13, there is no clear correlation between image reduction and density values. Instead, abrupt variations and localized concentrations of points are observed. This irregularity can be attributed to several factors: first, the neural rendering process in NeRF tends to extrapolate and infer geometry from learned visual priors, which becomes increasingly unstable with limited image input. Second, as more images are removed, the exported models begin to display gaps and artificial voids in regions that should be continuous, while simultaneously generating point clusters in erroneous areas—an effect clearly visible in the Nerfacto mesh visualizations that follow.

These results demonstrate that iTwin Capture Modeler offers superior consistency and reliability in processing time, geometric fidelity, and structural completeness, especially under conditions of reduced image availability. Nerfacto, by contrast, excels in visual realism, but its dependence on dense image input and its susceptibility to geometric inaccuracies make it more suitable for exploratory visualization or communication purposes rather than precision modeling.

The mesh outputs generated with Nerfacto were analyzed to assess their geometric completeness and structural consistency across the ten experimental datasets. These meshes, derived from the previously exported point clouds, revealed a notable sensitivity to the volume and quality of the input image data. In experiments using 100% to 60% of the original dataset, the resulting meshes exhibited continuous surfaces and recognizable architectural features, although some geometric artifacts and surface irregularities were present, particularly in complex or occluded areas.

As the number of images decreased below 50%, the meshes began to display more significant deficiencies. Large voids appeared in areas that should be structurally solid, while other regions showed implausible surface geometries or disconnected elements. These issues suggest that Nerfacto, when operating with reduced datasets, tends to concentrate points in localized zones while neglecting others, likely due to insufficient multi-view coverage and overfitting during volumetric inference. Consequently, while Nerfacto can produce visually rich reconstructions, its mesh generation process lacks the robustness required for metric reliability under constrained imaging conditions. Figure 7, Figure 8, Figure 9, Figure 10, Figure 11 and Figure 12 also illustrates the progressive degradation in mesh integrity as image input is reduced, reinforcing the limitations of NeRF-based models for accurate geometric reconstruction when data availability is limited.

## 5. Discussion

The comparative analysis between NeRF and photogrammetry reveals distinct strengths and limitations in their application to 3D modeling of civil infrastructure. Each method offers valuable advantages depending on the context of use, data availability, and the specific objectives of the modeling task.

From a technological standpoint, NeRF demonstrates notable versatility and exceptional visual quality. Its ability to generate photorealistic models with relatively fewer input images makes it attractive for scenarios where rapid visualization or immersive communication is prioritized—such as stakeholder presentations, preliminary inspections, or virtual reality environments. However, these benefits come with limitations. The lack of inherent scale, the instability under reduced image sets, and the difficulties in exporting dense, metrically accurate models make NeRF less suitable for analytical or design-driven engineering tasks. Moreover, the process of training neural networks is computationally intensive and does not scale linearly with data reduction, as evidenced in the fixed processing times across experiments.

In contrast, photogrammetry offers high geometric accuracy and consistent structural completeness, especially when high image overlap and precise metadata are available. It remains the standard for applications requiring engineering-grade measurements, such as structural diagnostics, survey-grade documentation, and deformation monitoring. However, photogrammetry is more sensitive to image quality and quantity, requiring larger datasets, well-planned acquisition protocols, and longer processing times, particularly for complex or large-scale scenes.

These complementary characteristics suggest distinct practical applications in civil engineering. NeRF may be better suited for contexts where visual communication is key and geometric precision is secondary—for example, heritage visualization, progress monitoring in construction, or community engagement with urban projects. On the other hand, photogrammetry remains the method of choice for detailed engineering analysis, topographic surveys, inspection of existing structures, and as-built documentation. These use cases are summarized in Table 13, which outlines specific engineering scenarios and the recommended technology based on performance criteria.

Importantly, the findings also highlight the potential for hybrid workflows, leveraging the strengths of both approaches. For example, a NeRF model could be used to quickly preview a scene or identify areas of interest before deploying more resource-intensive photogrammetric reconstruction. Conversely, NeRF-generated views could assist in gap filling or enhance the visual clarity of photogrammetric outputs when texture data is poor. This hybridization is particularly promising in constrained environments—such as inaccessible sites or emergency response situations—where capturing fewer images is necessary but visualization and structural understanding remain critical.

**Figure 14:**
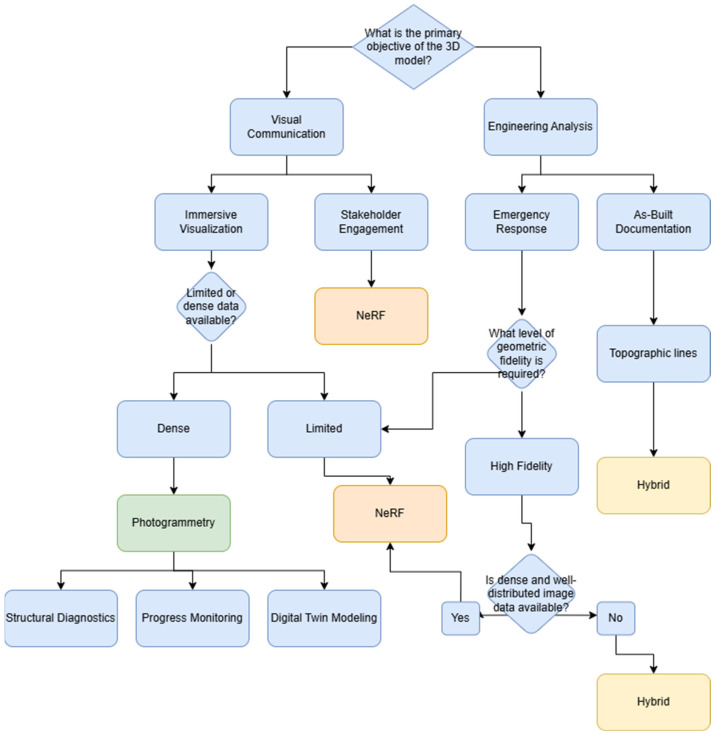
Decision Flowchart for Technology Selection in Civil Engineering Contexts.

Figure 14 presents the decision-support flowchart designed to guide the selection of appropriate 3D modeling technology in civil engineering projects, based on the intended purpose of the model, the nature of the available data, and the specific technical requirements of the application. The diagram begins by prompting the user to define the primary objective of the 3D model, offering two main branches, Visual communication and Engineering analysis, because this initial distinction reflects the fundamental divergence in modeling priorities—whether the focus lies on photorealistic rendering and user engagement or on accurate spatial measurements and technical evaluation. This bifurcation ensures that the selection process is tailored to the end-use requirements from the outset.

On the visual communication side, the flow splits into use cases such as stakeholder engagement and immersive visualization, where the emphasis lies in photorealistic rendering rather than metric accuracy. The next decision node assesses data availability, distinguishing between limited and dense datasets. If data is limited—typical of time-constrained or inaccessible environments—NeRF is recommended for its ability to produce visually coherent models with fewer images. If the dataset is dense, photogrammetry is preferred due to its ability to fully exploit high-resolution image sets for more detailed reconstruction. Both paths may lead to downstream applications such as progress monitoring and digital twin modeling, where NeRF offers rapid deployment and visual clarity.

On the engineering analysis side, the flow addresses more technical and precision-dependent scenarios such as emergency response and as-built documentation. The user is then asked whether hybrid or photogrammetry-based modeling is preferred. If high metric accuracy is required and sufficient image coverage is available, photogrammetry is advised for tasks like structural diagnostics and topographic surveys. If flexibility or partial modeling is acceptable due to environmental constraints, a hybrid workflow is recommended, combining NeRF for fast visualization with photogrammetry applied selectively to key structural elements.

The flowchart ultimately converges on three decision outcomes: exclusive use of NeRF, photogrammetry, or a hybrid workflow, each matched to the demands of the scenario. This visual decision logic encapsulates the complementary strengths of both technologies and highlights their optimal integration under varying field and project conditions.

## 6. Conclusions

This study addresses a critical research gap in civil engineering: the lack of empirical, side-by-side evaluations of Neural Radiance Fields (NeRFs) and digital photogrammetry under conditions of limited image availability. In real-world field operations, constraints such as restricted access, adverse weather, and limited flight time often lead to reduced image datasets. In this context, the central engineering challenge is not visual realism alone, but the ability to obtain geometrically reliable models with minimal acquisition effort. Despite the growing interest in NeRF-based reconstruction techniques and the maturity of photogrammetry, few studies have systematically examined how these methods behave when image availability is progressively reduced from an operational perspective.

To address this gap, a comparative analysis was conducted using a real-world case study and structured within the Design Science Research Methodology (DSRM). Although visual quality was considered, the primary evaluation criterion of this study was geometric accuracy, assessed through quantitative comparisons between dimensional measurements extracted from the reconstructed 3D models and reference measurements obtained directly from the physical structure. Additional metrics included point cloud density and processing time.

A key methodological contribution of this work is the implementation of a progressive image reduction strategy motivated by survey efficiency and cost considerations. Starting from an initial dataset of 475 UAV images, the number of images was reduced in 10% increments to simulate increasingly economical acquisition campaigns. This approach reflects a common engineering trade-off: fewer images enable wider coverage, shorter flight times, and reduced field and processing costs, but may affect geometric reliability.

The results demonstrate that, while photogrammetry exhibits a relatively stable error trend as image availability decreases, NeRF-based reconstruction consistently achieves lower average geometric error across most experimental conditions, as shown in Figure 6. This indicates that NeRF can maintain geometrically usable models even when the number of input images is significantly reduced, with noticeable degradation occurring only in the most constrained scenarios. From an engineering standpoint, this suggests that NeRF-based approaches can support more economical survey designs without immediately compromising dimensional reliability.

At the same time, photogrammetry continues to provide highly interpretable, metrically consistent point clouds, making it a robust solution when strict geometric completeness and direct CAD/BIM integration are required. However, its reliance on larger image datasets and longer processing times reduces flexibility in time- or resource-constrained field operations.

These findings support the adoption of hybrid workflows, where NeRF can be used to enable rapid, cost-efficient data acquisition and preliminary geometric assessment, while photogrammetry can be applied selectively when higher geometric completeness or downstream engineering integration is required. The proposed evaluation framework and decision matrix offer practical guidance for selecting reconstruction technologies based on geometric accuracy requirements, acquisition cost, and operational constraints, rather than visual fidelity alone.

Future research should extend this work by incorporating additional real-world degradation factors—such as illumination variability, shadow occlusion, motion blur, and restricted viewpoints—within controlled experimental designs that allow their individual and combined effects to be evaluated without obscuring the relationship between image availability, acquisition cost, and geometric accuracy. Such studies would complement the present results and help establish a more comprehensive understanding of NeRF and photogrammetry performance across the full spectrum of limited-data scenarios encountered in engineering practice.

## Figures and Tables

**Table 1:** Design Science Research Methodology (DSRM).

Stage	Goal	Main Activities	Tools
1. Problem Identification	Understand current uses of NeRF in 3D modeling and its potential in civil engineering	Literature review Identify relevant NeRF models and features	Systematic literature review
2. Objective Definition	Define criteria for comparing NeRF and photogrammetry (accuracy, visual quality, processing time)	Set evaluation metrics Design UAV flight plan for data acquisition	UAV survey planning
3. Artifact Design	Develop workflow for model creation in NeRF (Nerfacto) and photogrammetry (iTwin Capture Modeler)	Define image processing workflow Establish experimental setup	Nerfstudio, iTwin Capture Modeler
4. Implementation	Apply the workflow under varying data availability conditions	Capture and reduce image sets Generate NeRF and photogrammetry models	DJI Air 2S, Nerfstudio, iTwin Capture Modeler
5. Evaluation	Assess model performance and utility for civil engineering tasks	Compare processing time, accuracy, point clouds Visualize and quantify results	Nerfstudio, CloudCompare

**Table 2:** Number of images for each experiment.

Exp. 1 (100%)	Exp. 2 (90%)	Exp. 3 (80%)	Exp. 4 (70%)	Exp. 5 (60%)	Exp. 6 (50%)	Exp. 7 (40%)	Exp. 8 (30%)	Exp. 9 (20%)	Exp. 10 (10%)
475	429	382	334	286	240	189	141	93	46

**Table 3:** Results of experiment #1 (Approximately 250 million points).

Application	Exported Point Clouds		Processing Times
iTwin Capture Modeler	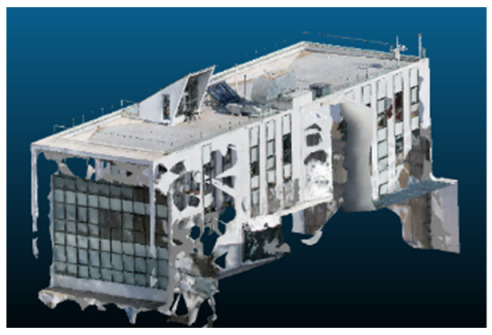	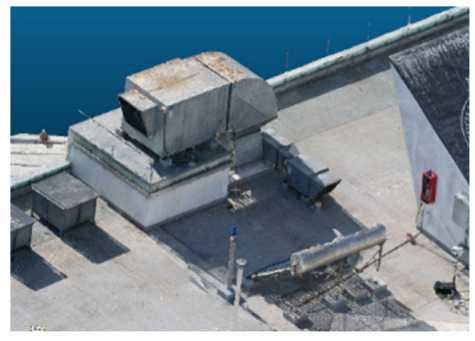	Aerial triangulation = 18 m Point cloud generation = 1:5 h
Nerfstudio	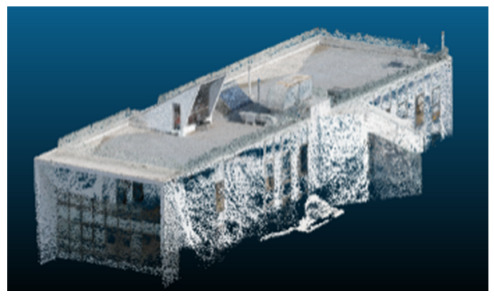	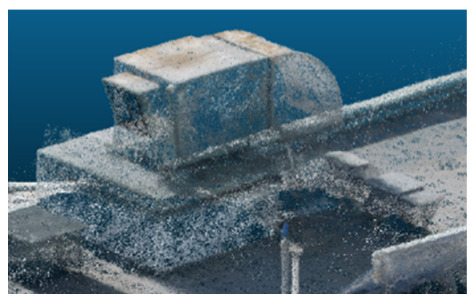	Camera alignment 9 m Model generation 28 m Point cloud export = 1:40 h

**Table 4:** Results of experiment #2 (Approximately 240 million points).

Application	Exported Point Clouds		Processing Times
iTwin Capture Modeler	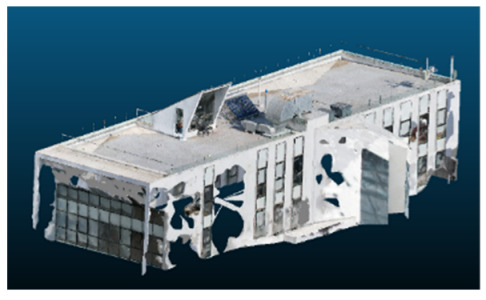	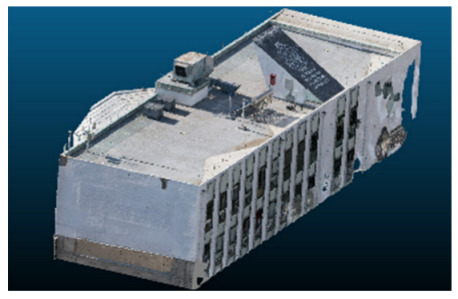	Aerial triangulation 15 m Point cloud generation = 1:38 h
Nerfstudio	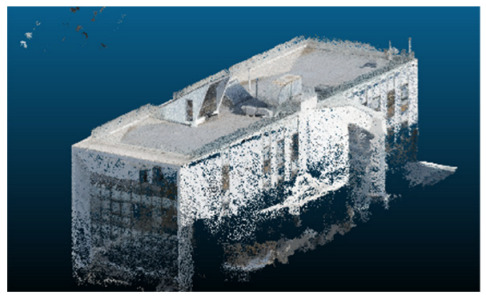	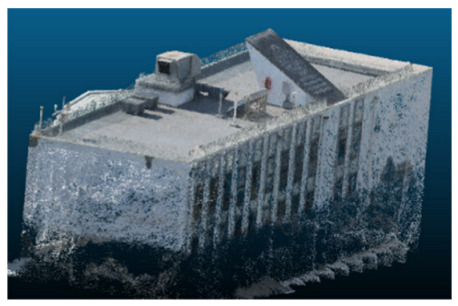	Camera alignment 8 m Model generation 28 m Point cloud export = 1:35 h

**Table 5:** Results of experiment #3 (Approximately 230 million points).

Application	Exported Point Clouds		Processing Times
iTwin Capture Modeler	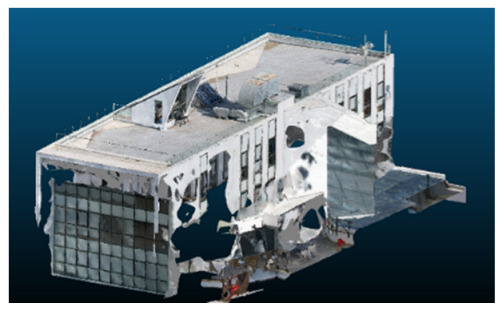	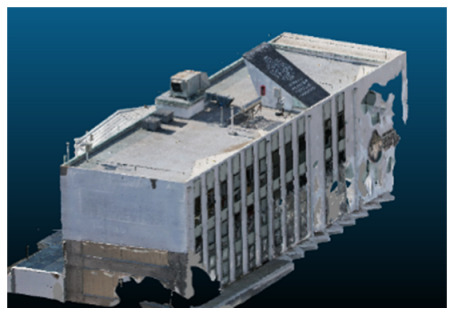	Aerial triangulation 13 m Point cloud generation = 1:21 h
Nerfstudio	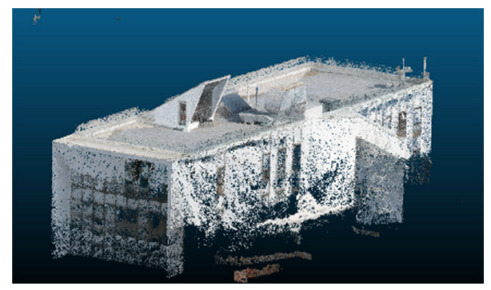	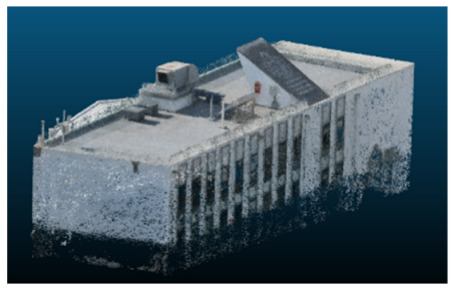	Camera alignment 8 m Model generation 28 m Point cloud export = 1:30 h

**Table 6:** Results of experiment #4 (Approximately 230 million points).

Application	Exported Point Clouds		Processing Times
iTwin Capture Modeler	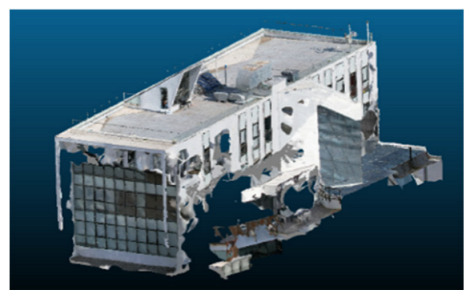	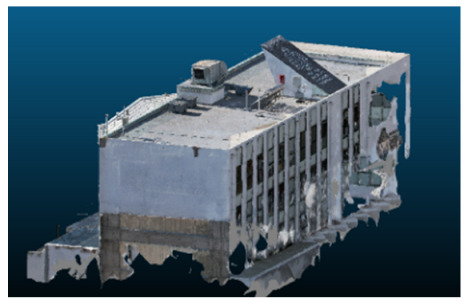	Aerial triangulation 11 m Point cloud generation = 1:29 h
Nerfstudio	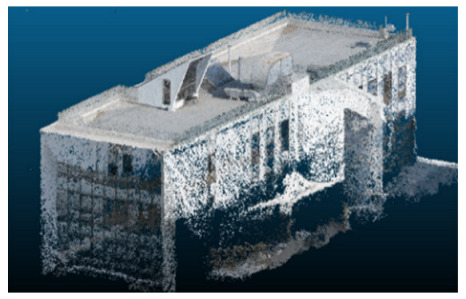	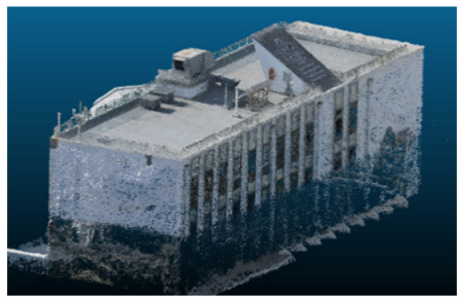	Camera alignment 7 m Model generation 28 m Point cloud export = 1:30 h

**Table 7:** Results of experiment #5 (Approximately 220 million points).

Application	Exported Point Clouds		Processing Times
iTwin Capture Modeler	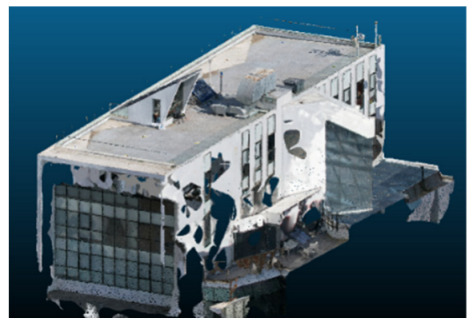	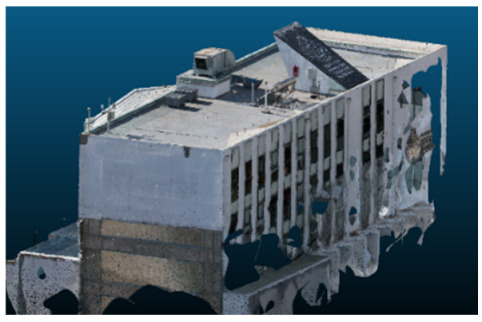	Aerial triangulation = 9 m Point cloud generation = 58 m
Nerfstudio	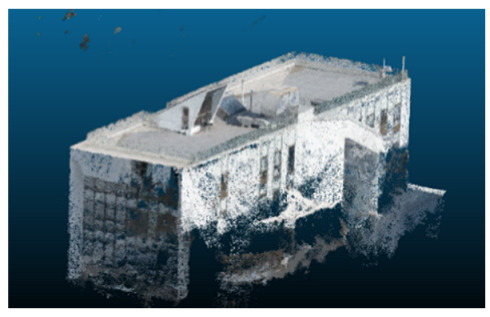	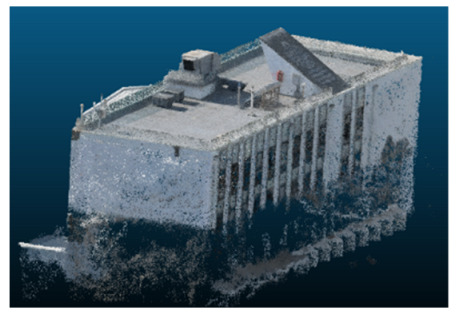	Camera alignment = 5 m Model generation = 28 m Point cloud export = 1:20 h

**Table 8:** Results of experiment #6 (Approximately 210 million points).

Application	Exported Point Clouds		Processing Times
iTwin Capture Modeler	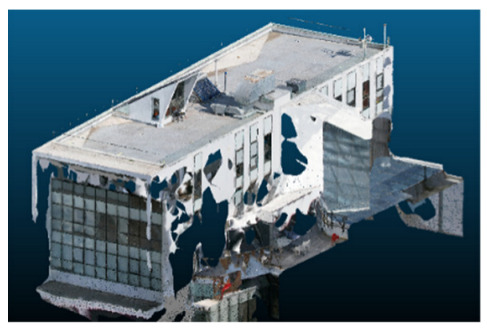	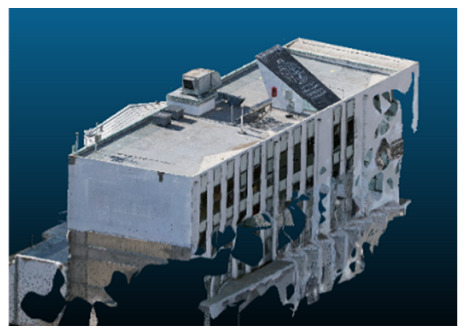	Aerial triangulation 8 m Point cloud generation = 48 m
Nerfstudio	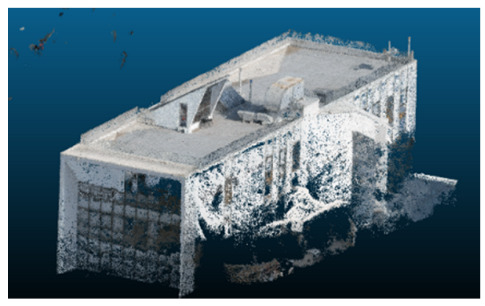	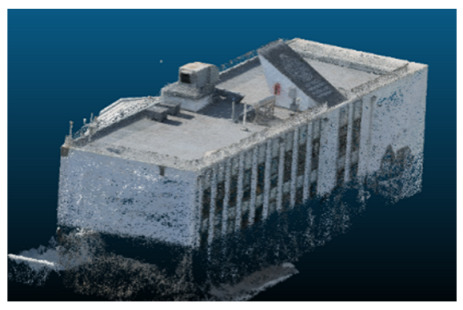	Camera alignment 4 m Model generation 28 m Point cloud export = 1:15 h

**Table 9:** Results of experiment #7 (Approximately 205 million points).

Application	Exported Point Clouds		Processing Times
iTwin Capture Modeler	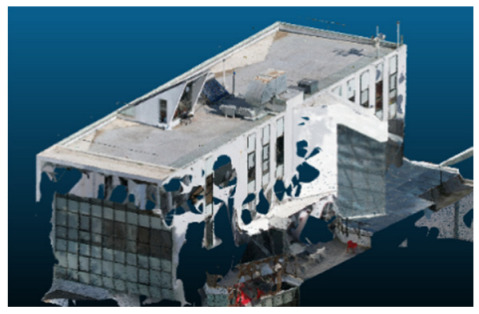	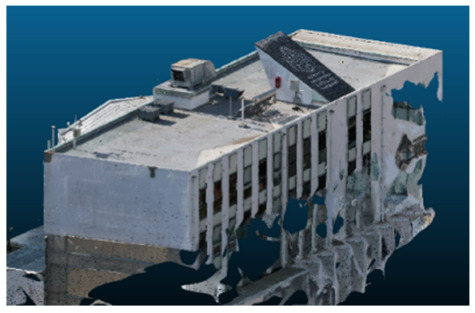	Aerial triangulation = 7 m Point cloud generation = 40 m
Nerfstudio	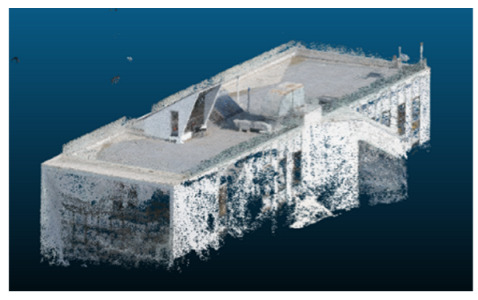	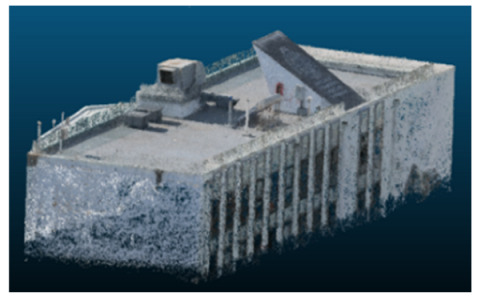	Camera alignment = 3 m Model generation = 28 m Point cloud export = 1:05 h

**Table 10:** Results of experiment #8 (Approximately 165 million points).

Application	Exported Point Clouds		Processing Times
iTwin Capture Modeler	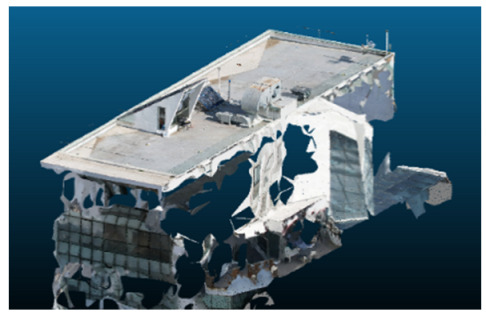	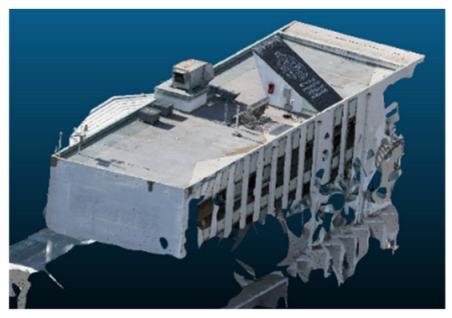	Aerial triangulation = 5 m Point cloud generation = 24 m
Nerfstudio	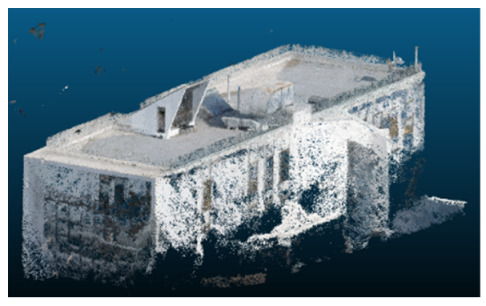	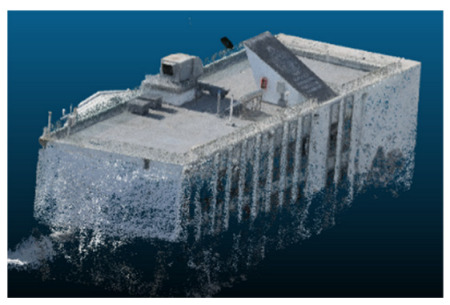	Camera alignment = 2 m Model generation = 28 m Point cloud export = 50 m

**Table 11:** Results of experiment #9 (Approximately 140 million points).

Application	Exported Point Clouds		Processing Times
iTwin Capture Modeler	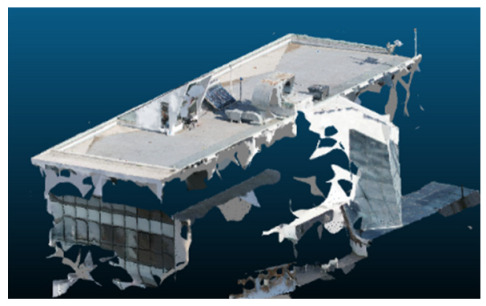	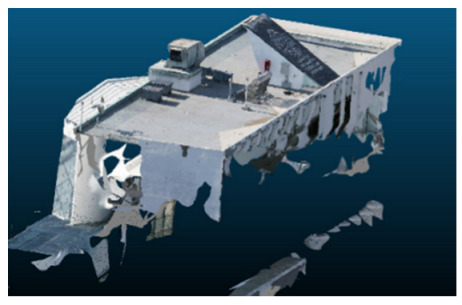	Aerial triangulation = 3 m Point cloud generation = 17 m
Nerfstudio	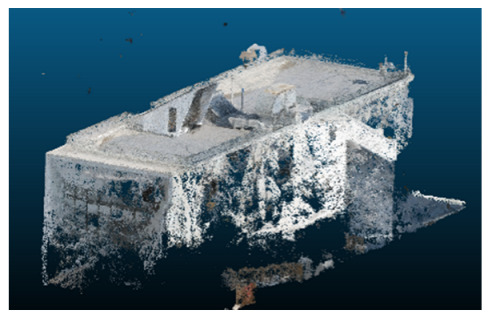	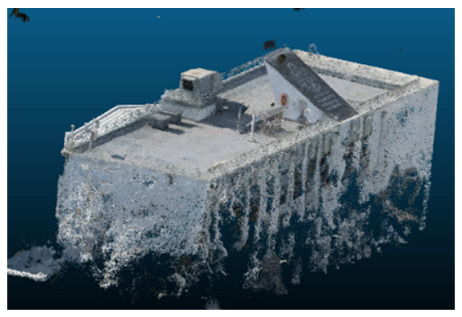	Camera alignment = 2 m Model generation = 28 m Point cloud export = 40 m

**Table 12:** Results of experiment #10 (Approximately 105 million points).

Application	Exported Point Clouds		Processing Times
iTwin Capture Modeler	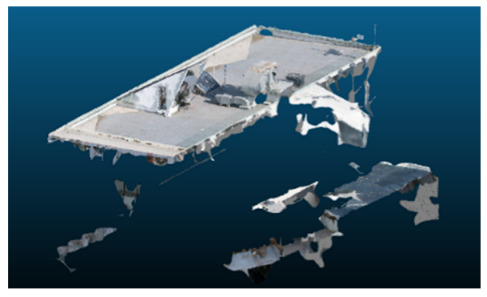	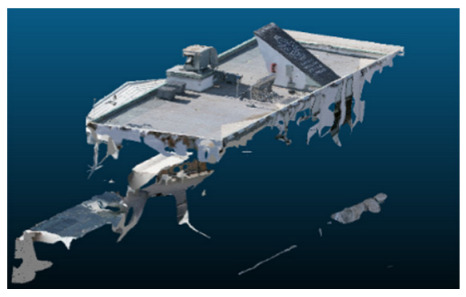	Aerial triangulation 2 m Point cloud generation = 10 m
Nerfstudio	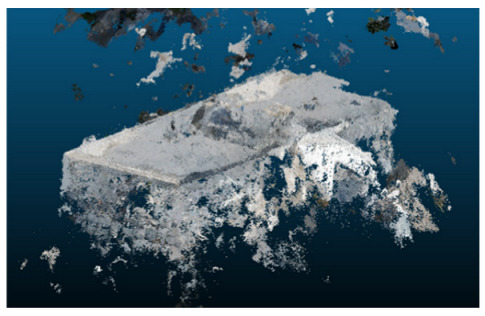	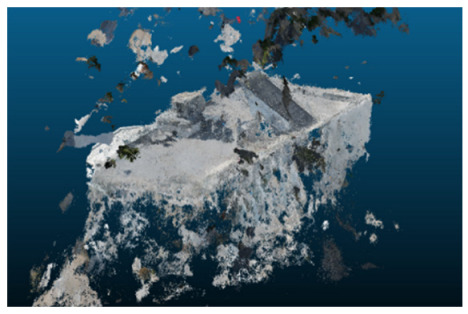	Camera alignment 1 m Model generation 28 m Point cloud export = 30 m

**Table 13:** Comparative Applications of NeRF and Photogrammetry in Civil Engineering.

Application Context	Recommended Technology	Justification
Visual inspection/immersive visualization	NeRF	High photorealism with fewer images; ideal for communication with stakeholders or early assessments.
Structural diagnosis/dimensional analysis	Photogrammetry	Delivers accurate scale and geometry; suitable for deformation tracking and precise measurements.
Heritage documentation	Hybrid (NeRF + Photogrammetry)	NeRF provides visual clarity; photogrammetry ensures metric fidelity for conservation modeling.
Progress monitoring in construction	NeRF	Fast model generation and ease of interpretation for frequent updates.
As-built documentation	Photogrammetry	Generates georeferenced, metrically accurate models for BIM and regulatory records.
Emergency response/inaccessible environments	NeRF	Operates effectively with reduced data; faster deployment when full capture is not feasible.
Digital twin or smart city modeling	Hybrid (NeRF + Photogrammetry)	Combines visual fidelity (NeRF) and dimensional precision (photogrammetry) for comprehensive models.

## Data Availability

The original contributions presented in this study are included in the article. Further inquiries can be directed to the corresponding authors.
